# Combination therapy involving radiofrequency ablation and targeted chemotherapy with bevacizumab plus paclitaxel and cisplatin in a rabbit VX2 lung tumor model

**DOI:** 10.1186/s13104-018-3358-x

**Published:** 2018-04-24

**Authors:** Ai Ueki, Tomohisa Okuma, Shinichi Hamamoto, Ken Kageyama, Kazuki Murai, Yukio Miki

**Affiliations:** Department of Diagnostic and Interventional Radiology, Osaka City University Graduate School of Medicine, 1-4-3 Asahi-machi, Abeno-ku, Osaka, 545-8585 Japan

**Keywords:** Radiofrequency ablation, Rabbit, Lung, Chemotherapy, VX2 tumor

## Abstract

**Objective:**

Radiofrequency ablation (RFA) is less effective for large tumors > 3 cm in diameter. Various studies of combination therapy using RFA and other treatments have been conducted to improve the results of RFA treatment of lung tumors, survival was extended in a tumor model when RFA was followed by concomitant use of systemic chemotherapy. Bevacizumab (BCM) is a one of molecular target drugs. Numerous clinical trials and reports have shown BCM’s effect when used in combination with cisplatin (CDDP) in lung tumor. Our objective is to evaluate the survival of concurrent, combined use of radiofrequency ablation and BCM, and platinum-doublet chemotherapy [CDDP/paclitaxel (PTX)] in a rabbit VX2 lung tumor.

**Results:**

Survival times of the RFA alone, CDDP/PTX, CDDP/PTX/BCM, RFA/CDDP/PTX, and RFA/CDDP/PTX/BCM groups were significantly prolonged compared to that of the control group (P = 0.0055, P = 0.0055, P = 0.0004, P = 0.0002, P = 0.0019, respectively). Survival of the RFA/CDDP/PTX/BCM group was not significantly prolonged compared to the RFA alone (P = 0.53) and CDDP/PTX/BCM group (P = 0.68), while showing a significantly shorter survival time than that of the RFA/CDDP/PTX group (P = 0.017). The addition to BCM with combination RFA and systemic therapy with CDDP/PTX did not have a positive effect on survival.

## Introduction

Radiofrequency ablation (RFA) is a well-established procedure achieving local tumor control for small tumors. However, it is less effective for large tumors > 3 cm in diameter [1–3]. Various studies of combination therapy using radiofrequency ablation and other treatments have been conducted to improve the results of RFA treatment of lung tumors [4–12]. A limited number of combination therapies have been reported in experimental models [13–16].

Molecular target drugs are widely used in the field of chemotherapy for malignant tumors. One of such drugs is bevacizumab (BCM), a humanized monoclonal antibody that targets vascular endothelial growth factor (VEGF). Numerous clinical trials and reports have shown BCM’s effect when used in combination with cisplatin. Chemotherapy combining BCM with carboplatin/paclitaxel has been reported to be useful for non-small-cell lung cancer, and this combination is widely used in clinical practice [17–19].

One experimental study on RFA + BCM combination therapy found only a single study of nude mice with human hepatocellular carcinoma, and evaluated the blood flow distribution [20]. There appear to be no studies using RFA + systemic chemotherapy added to BCM to treat lung tumors. In an earlier study, a rabbit VX2 lung tumor model was subjected to RFA, followed by chemotherapy using cisplatin (CDDP)/paclitaxel (PTX), and survival was extended [15]. A combination of RFA with chemotherapy may increase intratumoral drug accumulation by increasing blood flow and membrane permeability and provide survival benefit. We hypothesized that the addition of an antiangiogenic drug to RFA with chemotherapy may inhibit VEGF signaling after ablation and reduce the healing response, which could prevent remaining tumor cells from re-establishing the tumor and prolong the survival period. Therefore, the purpose of this present study was to investigate the efficacy of adding BCM to combination RFA and chemotherapy in the rabbit VX2 lung tumor model.

## Main text

### Methods

All experiments were approved by the animal care and use committee. All procedures performed in studies involving animals were in accordance with the ethical standards of the institution or practice at which the studies were conducted. A total of 42 female Japanese white rabbits (Japan SLC, Hamamatsu, japan) weighting 2.0–2.5 kg of 12 weeks of age were used for the study.

The rabbits have free access to standard chow and tap water in in our laboratory animal center. General anesthesia was induced before all procedures (tumor implantation, RFA, anticancer drug administration) by intramuscular injection of a 1-mL solution containing 40 mg (0.8 mL) ketamine (Daiichi-Sankyo, Tokyo, Japan) and 4 mg (0.2 mL) xylazine (Bayer, Leverkusen, Germany) and was maintained during the procedure by injection of 0.1 mL of the same solution via an ear vein as necessary.

#### Experimental models

VX2 carcinoma cells (Japan SLC Inc., Hamamatsu, Japan) were used to establish a model of lung tumors. Isolation and implantation of the VX2 tumor (the only tumor established in rabbits) were performed as previously described [15, 16]. In brief, a suspension of single VX2 tumor cells (2.5 × 10^6^ cell/mL) was prepared, of which 0.2 mL were injected with a 20-gauge needle into the left lower lobes of the lungs of each of 69 rabbits using single helical computed tomography (CT) guidance with the following parameters: voltage 120 kV; current 60 mA; collimation 3 mm; slice thickness 3 mm; pitch 1 and field of view (FOV) 20 cm, ReconMatrix size 320 × 320 (ProSpeed; GE Healthcare, Milwaukee, WI, USA.). Establishment of the lung tumor was confirmed on CT as a nodule 3–9 mm in diameter (mean 5.8 ± 1.9 mm) at 1 week after implantation (week 1). Of the remaining 27 rabbits, which were excluded from the study, 7 showed pleural dissemination in the lung but no solid mass or distant metastases, and 20 showed no viable tumor cells at postmortem examination. Thus, 42 rabbits with established tumors were used for the experiments. The surviving rabbit treated with RFA/CDDP/PTX was euthanized via carbon dioxide aspiration after the complete study.

#### Study groups

A total of 42 experimental animals were randomly assigned to six experimental groups based on randomization tables (n = 7 each): a control group that was followed up without treatment (i.e., supportive care alone); an RFA group that underwent RFA of the lung tumor alone; two chemotherapy groups that received chemotherapy alone (either CDDP/PTX or CDDP/PTX/BCM); and two combination therapy groups (RFA/CDDP/PTX, RFA/CDDP/PTX/BCM) that received treatment with chemotherapy immediately after RFA. All treatment groups received treatment only once. All treatment groups were not administered antiemetic before receiving chemotherapy.

#### RFA

**Fig. 1:**
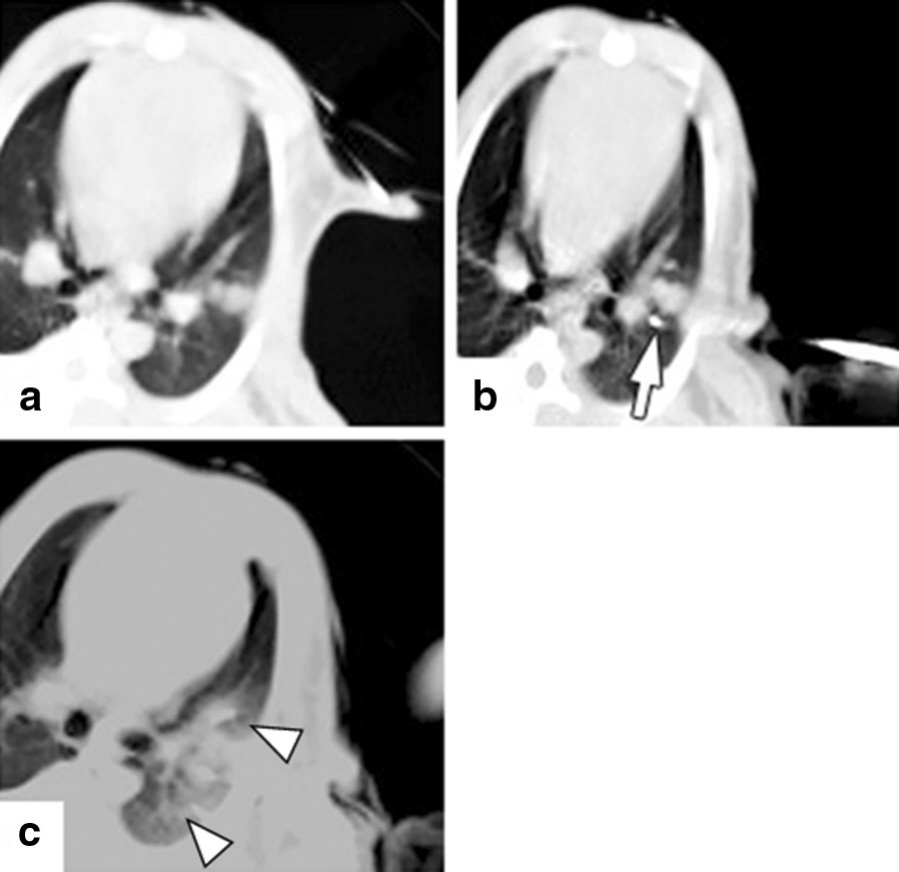
**a** CT image of VX2 tumor before RFA. A round VX2 tumor with a well-defined boundary is seen in the left lower lobe. **b** CT image of VX2 tumor during RFA. A LeVeen Needle is puncturing the tumor, and the electrode’s tip (arrow) has been expanded. **c** CT image of VX2 tumor immediately after RFA. A ground glass opacity (arrowhead) is observed around the tumor, confirming its ablation. The electrode’s tips are seen

Radiofrequency ablation was performed at week 1 by using a LeVeen electrode (Boston Scientific, Natick, MA, USA) with 8 retractable hooks having a maximum diameter of 2 cm and an RF2000 generator (Boston Scientific). The electrode was percutaneously inserted into the lung tumor under CT guidance. The retractable hooks were fully opened before ablation in all cases. Tumor ablation was performed at an output of 20 W and continued until the generator stopped automatically upon reaching the maximum resistance due to increased impedance (i.e., maximum impedance). CT scans were obtained after RFA to confirm that ground-glass opacity had appeared around the tumor (Fig. 1).

#### Anticancer drugs

Cisplatin was purchased from Nichi-Iko Pharmaceutical Co., Ltd. (Tokyo, Japan), PTX was purchased from Sawai Pharmaceutical Co., Ltd. (Osaka, Japan), and BCM was purchased from Chugai Pharmaceutical Co., Ltd. (Tokyo, Japan). Following systemic chemotherapy, cisplatin (CDDP) (2.5 mg/kg, 0.5 mg/mL) + paclitaxel (PTX) (7.5 mg/kg, 1.5 mg/mL), bevacizumab (BCM) (10 mg/kg, 1 mg/mL) was given. Anticancer drugs were injected slowly (over at least 30 min) into the auricular vein. In the combination therapy groups (i.e., RFA/CDDP/PTX, RFA/CDDP/PTX/BCM), they were given immediately once after RFA.

#### Statistical analysis

GraphPad Prism 6 software (GraphPad Software, Inc., La Jolla, CA, USA) was used for the statistical analysis. The Kaplan–Meier method with the log-rank test was used for end-point survival analysis. The interquartile range (IQR) was calculated to disclose data variance and/or the data distribution of median survival times. Differences with *P* values of less than 0.05 were considered significant. Median survival time was defined as the period from tumor implantation to death, with a maximum observation period of 120 days.

### Results

#### Survival

The median survival time was 26 days (range 21–28 days, IQR = 4 days) in the control group, 34 days (range 26–105 days, IQR = 26.5 days) in the RFA group, 30 days (range 26–56 days, IQR = 20 days) in the CDDP/PTX group, 38 days (range 28–44 days, IQR = 8.5 days) in the CDDP/PTX/BCM group, 120 days (range 36–120 days, IQR = 75.5 days) in the RFA/CDDP/PTX group, and 37 days (range 17–58 days, IQR = 10 days) in the RFA/CDDP PTX/BCM group. For the RFA/CDDP/PTX group, the Kaplan–Meier survival rate at the end of the follow-up period was 57.1%. Because survival never fell to 50%, the Kaplan–Meier median survival was not defined but was greater than the 120 days median follow-up period (range 36–120 days).

Survival times of the RFA alone, CDDP/PTX, CDDP/PTX/BCM, RFA/CDDP/PTX, and RFA/CDDP/PTX/BCM groups were significantly prolonged compared to that of the control group (*P *= 0.0055, *P *= 0.0055, *P *= 0.0004, *P *= 0.0002, *P *= 0.0019, respectively).

**Fig. 2:**
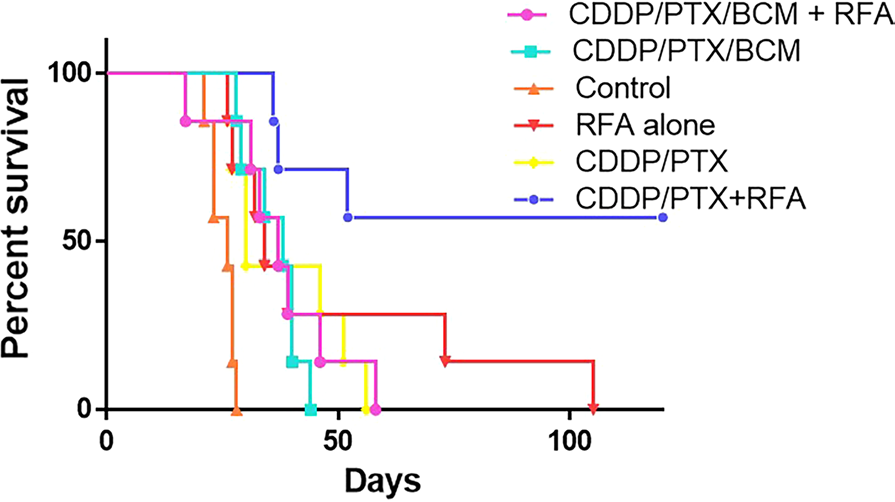
Survival times of the CDDP/PTX/BCM and RFA/CDDP/PTX/BCM groups are significantly prolonged compared to that of the control group. The CDDP/PTX/BCM group does not show significant differences in survival compared to the RFA alone group and the CDDP/PTX group. Survival of the RFA/CDDP/PTX group is significantly prolonged compared to that of the CDDP/PTX/BCM group. The survival time of the RFA/CDDP/PTX/BCM group is not significantly different from that of the RFA alone, and CDDP/PTX/BCM groups, but it is significantly shorter than that of the RFA/CDDP/PTX group

The CDDP/PTX/BCM group did not show a significant difference in survival compared to the CDDP/PTX group (*P *= 0.38). Survival of the RFA/CDDP/PTX group was significantly prolonged compared to that of the RFA alone (*P *= 0.022), and CDDP/PTX (*P *= 0.014). The survival time of the RFA/CDDP/PTX/BCM groups was not significantly different from that of the RFA alone (*P *= 0.52), CDDP/PTX (*P *= 0.81) and CDDP/PTX/BCM groups (*P *= 0.68). Survival of the RFA/CDDP/PTX/BCM group was significantly shorter than that of the RFA/CDDP/PTX group (*P *= 0.017) (Fig. 2).

### Discussion

Bevacizumab is an anti-VEGF antibody: it is a humanized monoclonal antibody that targets VEGF. BCM specifically binds to VEGF, as a result, BCM expresses its anti-tumor effects by directly inhibiting tumor angiogenesis, reducing interstitial pressure, and improving the vascular permeability that accompanies the normalization of tumor blood vessels, thereby improving the transfer of combination anticancer drugs to tumors [21, 22]. Various reports have shown synergistic effects when combining BCM with other antineoplastic drugs to treat non-small-cell lung cancer, colon cancer, breast cancer, and ovarian cancer [23–26].

To the best of our knowledge, two studies have investigated the effects of BCM on rabbit VX2 tumors. In one study [27], BCM was administered to VX2 tumors implanted in the back muscles, followed by examination of the early therapeutic effect, but the long-term course was not observed, which differs from the present study of post-treatment survival. In another study [28] on BCM administration in a VX2 carcinomatous meningitis model, the BCM treatment group showed significant prolongation of survival compared with the control group. In the present study, survival in the CDDP/PTX/BCM group (38 days) was significantly longer than in the control group (26 days), but there was no significant difference in survival between the CDDP/PTX/BCM (38 days) and CDDP/PTX (30 days) groups. Pascale et al. [29] evaluated the cytotoxic effect of various anticancer agents including BCM. They demonstrated that BCM’s drug sensitivity for the VX2 tumor cell line was poor, and even high concentrations of BCM had little effect [29]. The authors showed that BCM binds to VEGF, preventing the interaction of VEGF with its receptors, which are mostly distributed on endothelial cells, but has no direct cytotoxic effect on the VX2 tumor cells. While the endpoint is different between their cytotoxic effects after 72 h between our survival times, their results may be consistent with the present experimental finding that the addition of BCM was not effective.

The addition of an antiangiogenic drug to thermal ablation with chemotherapy may have three benefits: (1) the reduction in vascularity could reduce perfusion, thereby making thermal ablation more uniform [20]. (2) Vascular normalization could enhance drug delivery to the periphery of the ablated zone, thereby enhancing cell killing of any residual cells outside the ablated area. (3) Inhibition of VEGF signaling after ablation would reduce the wound healing response, which could prevent any other remaining tumor cells from re-establishing the tumor.

To the best of our knowledge, no study has investigated the effect of adding BCM to combination RFA and chemotherapy therapy on rabbit VX2 tumors. The only study that added RFA to BCM administration was performed by implanting hepatocellular carcinoma in nude mice and evaluating the treatment by power Doppler US after BCM administration [20]. They found that the tumor blood flow decreased after BCM administration, and they showed the usefulness of power Doppler US for evaluating efficacy. However, in that study, as well, long-term follow-up was not performed. The findings of this study were consistent with earlier study findings that the administration of CDDP/PTX after lung RFA was most effective against rabbit VX2 tumors [15], but the survival of adding BCM to combination RFA and CDDP/PTX was significantly shorter than that in the without BCM group [i.e., RFA/CDDP/PTX/BCM vs RFA/CDDP/PTX (37 vs 120 days)]. Thus, adding BCM to combination therapy involving lung RFA and systemic chemotherapy with CDDP/PTX may produce a negative effect. The anti-VEGF action of BCM causes decreased endothelial cell repair function, decreases in platelet aggregation and coagulation factors are thought to result in incomplete wound healing. BCM is also believed to cause central tumor necrosis and to enlarge the tumor cavity with immature blood vessels, those events may lead to complications. Studies on non-small cell lung carcinoma, renal cancer, colorectal cancer reported cases of high-grade bleeding associated with BCM-combined chemotherapy, and the risk with that therapy was said to be high [30–32].

### Conclusions

The addition of BCM to combination RFA and systemic chemotherapy with CDDP/PTX did not have a positive effect on survival compared to RFA/CDDP/PTX for treatment in a rabbit lung cancer model with VX2 tumor.

## Limitations

The present study has several limitations. Firstly, the study was conducted in a rabbit model. Although simultaneous lung RFA and chemotherapy with BCM may be considered feasible in human patients, the VX2 tumor may exhibit different treatment responses and is considered more malignant than human lung tumors. Secondly, the schedule of chemotherapy: we followed earlier studies of chemotherapy performed immediately after RFA for VX2 lung model [15], but it may be clinically relevant to give anticancer drugs prior to RFA. Thirdly, tumor response was not evaluated longitudinally by CT, because general anesthesia is necessary in animals during CT scanning, and it may affect survival. In addition, it is difficult to evaluate the treatment response by CT at the early stages after RFA. For these reasons, survival was the primary endpoint. Lastly, the present study did not include a histopathological examination, since histopathological changes of lung VX2 tumors after RFA have been previously studied [14, 15]. But, further study will be necessary to confirm the histological changes when adding to BCM with combination RFA and chemotherapy.

## Abbreviations

RFA: radiofrequency ablation
BCM: bevacizumab
VEGF: vascular endothelial growth factor
CDDP: cisplatin
PTX: paclitaxel
CT: computed tomography
FOV: field of view
IQR: interquartile range
